# Mass production of highly-porous graphene for high-performance supercapacitors

**DOI:** 10.1038/srep32686

**Published:** 2016-09-08

**Authors:** Ahmad Amiri, Mehdi Shanbedi, Goodarz Ahmadi, Hossein Eshghi, S. N. Kazi, B. T. Chew, Maryam Savari, Mohd Nashrul Mohd Zubir

**Affiliations:** 1 Department of Mechanical Engineering, University of Malaya, Kuala Lumpur, Malaysia; 2 Department of Chemical Engineering, Faculty of Engineering, Ferdowsi University of Mashhad, Mashhad, Iran; 3 Department of Mechanical and Aeronautical Engineering, Clarkson University, Potsdam, NY 13699, USA; 4 Department of Chemistry, Faculty of Science, Ferdowsi University of Mashhad, Mashhad, Iran; 5 Faculty of Computer Science and Information Technology, University of Malaya, Kuala Lumpur, Malaysia

## Abstract

This study reports on a facile and economical method for the scalable synthesis of few-layered graphene sheets by the microwave-assisted functionalization. Herein, single-layered and few-layered graphene sheets were produced by dispersion and exfoliation of functionalized graphite in ethylene glycol. Thermal treatment was used to prepare pure graphene without functional groups, and the pure graphene was labeled as thermally-treated graphene (T-GR). The morphological and statistical studies about the distribution of the number of layers showed that more than 90% of the flakes of T-GR had less than two layers and about 84% of T-GR were single-layered. The microwave-assisted exfoliation approach presents us with a possibility for a mass production of graphene at low cost and great potentials in energy storage applications of graphene-based materials. Owing to unique surface chemistry, the T-GR demonstrates an excellent energy storage performance, and the electrochemical capacitance is much higher than that of the other carbon-based nanostructures. The nanoscopic porous morphology of the T-GR-based electrodes made a significant contribution in increasing the BET surface as well as the specific capacitance of graphene. T-GR, with a capacitance of 354.1 Fg^−1^ at 5 mVs^−1^ and 264 Fg^−1^ at 100 mVs^−1^, exhibits excellent performance as a supercapacitor.

Traditionally, batteries are high-energy-storage devices with a poor-energy-delivery capability (e.g., low power density, low rate capability), whereas supercapacitors are low energy-storage devices but with a superior-energy-delivery capability. Hence, supercapacitors are considered to be great complement for batteries in different high-rate applications, such as high performance portable electronics and automobiles^1^. The nature and microstructure of the electrode materials are crucial for not only energy and power densities but also for safety and cycle lifetime of both batteries and supercapcitors^2^. Traditional carbon materials (e.g., carbon black, carbon nanofibers, graphite) and metal oxides have been intensively investigated as the electrode materials in both supercapcitors and batteries, with a recent focus on graphitic carbon materials to overcome some main weaknesses (e.g., poor electrical conductivity, huge charging-/discharging-induced volume change) associated with the traditional electrode materials^3^. As a promising material, the two-dimensional (2D) single atomic carbon sheet of graphene has emerged as an attractive candidate for energy applications due to its unique structure and properties^4^,^5^,^6^,^7^,^8^,^9^. Fully exploiting the properties of graphene with large specific surface area as electrode materials require a method for the mass production of this remarkable material. Two main routes are possible: large-scale growth or large-scale exfoliation.

As a large-scale growth method, chemical vapor deposition (CVD) technique has been recently used to grow thin and highly crystalline graphitic layers atop catalysts^10^,^11^. To achieve the goal of growing graphene, a volatile carbon precursor, such as methane, is exposed to a reductive media, subsequently decomposing on metal catalysts, such as Ni^12^, that perform in high temperature conditions. The epitaxial growth method in the presence of the annealing of SiC at high temperatures of about 2000 °C can fabricate high-quality graphene, which is deposited directly on the SiC wafer. This method is referred to as chemical solid deposition (CSD)^13^,^14^,^15^ since the precursor is a solid rather than a gas. Although the CVD and CSD approaches can be considered as the dominant growth methods for manufacturing high-quality graphene for future high-performance microelectronics applications, the industrially-scalable production of graphene with just a few layers with these approaches will be problematic.

As a large-scale exfoliation method, the production of chemically converted graphene from the reduction of graphene oxide is a convenient method to obtain large amounts of graphene^16^,^17^; however, even with efficient reducing agents such as hydrazine or H_2_, and annealing at high temperature, the original crystalline structure of graphene is not restored. Graphene oxide is heavily functionalized with many permanent chemical defects, such as holes, introduced into the basal plane. These holes are not readily healed even upon annealing^16^,^18^,^19^. Also, while thermal annealing can be considered as one of the best methods to heal some of holes, it does not seem to be cost-effective^20^.

As a novel technique to overcome the drawback with above-mentioned, the liquid phase exfoliation of graphite in the presence of high surface-tension organic solvents, along with continuous sonication^21^,^22^, opened a new gateway to achieve single-layered and/or sheets of graphene with just a few layers. Nevertheless, due to the lack of easily-miscible functional groups, such as polymers to decrease interlayer attractions, the graphene sheets suspended in the high-surface tension base fluids tend to aggregate^23^. Also, due to the considerable π–π interactions, liquid-phase exfoliated graphene cannot reach stable dispersion in base fluids, implying high level of aggregation^20^. As a novel method, liquid-phase exfoliation of graphite produces high-quality graphene without structural defects. However, due to lack of graphene-solubility, the exfoliation performances of most of the solvents suggested in previous attempts were quite low. Also, in some cases, the obtained samples are only partially exfoliated and still contain extensive domains of staked graphitic layers. To increase the efficiency of exfoliation with this method, the chemical functionalization of graphite with different functional groups, such as 4-bromophenyl, provides a new approach for improving its solubility in polar, aprotic, organic solvents, allowing the exfoliation of bulk graphite with fewer problems^20^,^24^.

For example, Sun *et al*.^20^ concluded that graphite can be changed to graphene by covalent functionalization of the graphite and the synthesis of a stable suspension in the presence of DMF without any added surfactant or stabilizer. Also, they achieved both exfoliation and functionalization with a fast procedure and obtained suitable edge-functionalization and intact pristine graphene structure in the interior basal planes. In addition, the morphological results showed that more than 70% of the graphene flakes had layers with thicknesses less than 5 nm, achieving a high yield of soluble graphene^20^.

To attain high yield of soluble graphene, graphite graft copolymers can be synthesized by covalent functionalization with the monofunctional groups to increase the dispersibility and wettability of the individual graphite and expanding flakes. The monofunctional group, as a covalent group, prevents cross-linking of the graphite flakes^25^. The functionalization of graphite with monofunctional group has resulted in graphite graft copolymers with high dispersibility in different media, such as DMF, DMA, g-butyrolactone (GBL), and ethylene glycol (EG), which can provide a promising approach for the fast and economical production of highly-porous, single-layered graphene. Also, functionalization with monofunctional groups can expand the graphite layers in EG with a surface tension of 47.70 mJ m^−2^ at 20 °C, it can be used to ensure appropriate conditions for exfoliation under mild sonication.

In this study, we report a fast exfoliation approach (far faster than the normally employed) to produce graphenes. First, graphite was functionalized predominantly with tetrahydrofurfuryl polyethylene glycol (PEG) using the *in situ* formation of an electrophilic addition reaction using microwave irradiation. The microwave-assisted approach was introduced for the functionalization of graphite via PEG in order to improve its dispersibility in polar, aprotic, organic solvents. By mild sonication in ethylene glycol (EG), chemically-assisted exfoliated, highly-porous, single-layered graphene (CE-GR) sheets were obtained from the bulk functionalized graphite. Interestingly, CE-GR sheets are significantly more stable in different solvents than pristine graphite. Thermal treatment was used to prepare pure graphene without any functional groups, and the pure graphene obtained in this way was labeled as T-GR. Results suggest that almost 84% of the T-GR flakes had just one layer, and 96% of the flakes had one and two layers. Regarding the number of layers, the exfoliation efficiencies of CE-GR and T-GR were fairly high and successful. In terms of electrochemical energy, the CE-GR and T-GR materials had high electrochemical capacitance. The T-GR materials with nanoscopic porous morphology are of high specific surface area (1559 m^2^/g (S_BET_) based on BET analyses of nitrogen cryo-adsorption method) and electrochemical capacitance up to 354 F g^−1^ (for the tenth cycle).

## Materials and Methods

### Microwave-assisted functionalization and *in situ* exfoliation

Figure S1 shows the experimental procedure for functionalization, as well as the exfoliation of graphite to produce CE-GR and T-GR. Regarding functionalization, in a typical experiment, the pristine graphite (10 mg) and 185.4 mg of AlCl_3_ as a Lewis acid were ball-milled and poured into a Teflon reaction vessel and 200 ml of PEG were gradually added during sonication for 30 min at room temperature to obtain a homogeneous suspension under nitrogen atmosphere. A special white smoke was seen during adding the PEG. Also, concentrated hydrochloric acid (0.5 mL) was added dropwise to the graphite suspension during the sonication process. Then, the mixture was poured into a Teflon vessel, sealed and transferred into an industrial microwave and irradiated at 150 °C at an output power of 700 W for 20 min (with frequently of 5 min sonication and 5 min placed under microwave irradiation). An electrophilic addition reaction occurred between the PEG and the graphite, resulting in the attachment of the PEG groups and hydroxyl groups to the exposed edges and side of the expanded graphite flakes, thereby producing functionalized, expanded graphite. Microwave irradiation was used to increase the speed of the reaction and the functionalization yield.

With a Lewis acid (AlCl_3_) as a catalyst and trace concentrated hydrochloric acid to protonate alcohols, electrophilic addition reactions were carried out between PEG and graphene through a microwave-induced method. A primary alcohol in the presence of Lewis acids and concentrated hydrochloric acid can be protonated and resulted in an electrophilic species (carbocation or protonated alcohol). This electrophile (in the case of poly ethylene glycol) is a stabilized cationic electrophilic reagent with significantly superior reactivity in the presence of microwave irradiation^26^,^27^. These types of carbocations were described by George Olah^28^,^29^. By protosolvation of a cationic electrophile, an active electrophilic reagent is suitable for electrophilic aromatic substitution, which in the case of the graphene flakes, an electrophilic addition reaction was occurred. The formed cations in the first step of electrophilic addition reaction on flakes reacts with nucleophiles (H_2_O, …)^28^,^29^.

Also, the reaction intensifies under microwave irradiation. When carbon nanostructures are exposed to microwaves, strong absorptions are obtained, which producing intense heating. Although the utilization of microwaves for the activation of carbon nanostructures has not been fully explored, the strong absorptions can open the door to the similar electrophilic addition reactions^26^,^30^. In addition, the edges of the expanded graphite were more available for reaction with the functional groups than the basal plane surfaces, which were stacked with strong π–π interactions. Note that the decomposition of the functional groups on functionalized flakes under microwave irradiation can also generate some pressure for overcoming the van der Waals interaction among graphene layers during the microwave procedure.

Subsequently, the resulting black ink-like dispersion was left to sit for 24 hr to separate large unstable graphite aggregates. The functionalized graphite without unstable graphite aggregates was expanded and was much more soluble in dimethylformamide (DMF), Dimethylacetamide (DMA), g-butyrolactone (GBL) and EG than the pristine graphite, as shown in Figure S2, panel 1. It is noteworthy that a majority of flakes remained stable for more than 1 week. Also, plot of absorbance versus wavelength for various colloidal solutions were studied to trace the presence of functionalized graphite within the binary system. Figure S3 show the plot of absorbance intensity versus wavelength and the colloidal stability of PEG-treated graphite in different solutions for expanded graphite in EG, DMA, DMF and GBL taken at specific period.

Then, the homogeneous suspension of PEG-treated graphite in EG was poured into another vessel that contained 450 ml of EG, and the mixture was sonicated for 2 hr to completely disperse the functionalized graphite flakes; this was followed by 30 min of centrifugation at 3000 rpm to collect the supernatant, which was filtered, washed, dried, and denoted as CE-GR after ball-milling the dry-powder for another 30 min. Part of the CE-GR was used for testing and the reminder was set aside for further treatment.

In order to obtain pure graphene without functional groups, thermal treatment up to 500 °C under a nitrogen atmosphere was applied for 15 min to remove all PEG molecules or other impurities. The product was labeled as T-GR. The pure graphene powder that was extracted was black (Figure S2 panel 2), which was a significant contrast with the shiny, metallic grey pristine graphite.

### Electrochemical Measurements

The electrochemical properties of CE-GR and T-GR are characterized by an organic system that includes MeEt_3_NBF_4_(AN) as an electrolyte and aqueous counterparts comprised of 6 M KOH. Also, the two-electrode system, shown in Figure S4 (Supporting Information), was used for the measurements. (Figure S4 shows a photograph of a coin-type test cell.) CV results in the scanning-rate range of 5 to 100 mV/s were obtained by Princeton PARSTAT 2273. An Arbin BT2000 tester was used to measure the Galvanostatic charge/discharge at different current densities.

## Results and Discussion

Graphene graft copolymers is first synthesized by covalent functionalization of graphite with the monofunctional, tetrahydrofurfuryl-terminated polyethylene glycol to increase the dispersibility and wettability of the individual graphite and expanding flakes. The monofunctional group, as a covalent group, prevents cross-linking of the graphite^25^. Also, we have followed another promising approach for the exfoliation of graphite to produce just highly-porous, single-layered graphene as a quick method for application to high-performance supercapacitors.

### Functionality and quality of graphene

As mentioned above, covalent functionalization occurs through the formation of an electrophilic addition reaction between graphite and the PEG chain under microwave irradiation, which was verified by FTIR spectroscopy.

**Figure 1:**
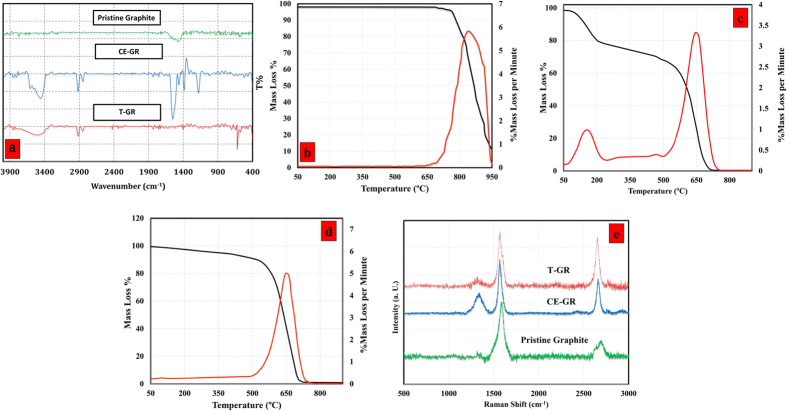
(**a**) FTIR spectra of pristine graphite, CE-GR and T-GR, TGA (black) and DTG (orange) curves of (**b**) pristine graphite, (**c**) CE-GR, (**d**) T-GR and (**e**) Raman spectra of pristine graphite, CE-GR and T-GR.

Figure 1(a) shows the FTIR spectra of pristine graphite, CE-GR, and T-GR. It is seen that the FTIR spectrum of pristine graphite provided no evidence of PEG. The peaks at 1471, 1535 and 1577 cm^−1^ were respectively consistent with the bending vibration of the CH_2_ group, stretching vibrations of the C=C and C=O. In contrast, the FTIR spectrum of CE-GR had two peaks in the range of 2800–3000 cm^−1^, which were associated with the C-H stretching vibration^31^. The peak at 1548 cm^−1^ was consistent with C=O or C=C stretching vibrations, which are infrared-activated by extensive functionalization. Also, the OH stretching vibration produced a weak peak at 3455 cm^−1^, indicating the presence of hydroxyl groups attached to the graphite. The FTIR spectrum of the treated samples also had a peak at 1460 cm^−1^, representing the bending vibration of the CH_2_ group. The peak at 1182 cm^−1^ was in agreement with the stretching vibration of the C–O groups. In contrast to the CE-GR, T-GR showed no evidence of PEG molecules, which confirmed that all of the functional groups had been removed. The FTIR spectrum of T-GR shows two weak peaks in the range of 2800–3000 cm^−1^, which were associated with the C-H stretching vibration. The lack of above-mentioned peaks in T-GR spectrum shows the prepared graphene was almost pure and that a majority of the functional groups had been removed^32^,^33^,^34^,^35^. To provide evidence supporting the above statements, thermogravimetric analysis (TGA) was used to detect quantitatively the weight fraction (loading) of organic groups attached to the graphite. Figure 1b–d (black curves) show the TGA curves of the pristine graphite, CE-GR, and T-GR samples, respectively. Also, Fig. 1b–d show the differential thermogravimetric analysis (DTG) curves (orange curves) of the pristine graphite, CE-GR, and T-GR powders after filtration. It is apparent that there was no significant weight loss with pristine graphite, which was thermally stable when heated to 770 °C under an air flow rate of 50 cm^3^/min. The DTG curves also show that there were two phases of degradation with the CE-GR sample. CE-GR illustrates a mild weight loss in the temperature range of 100–205 °C, which was due to the decomposition of the covalently-grafted organic addends (PEG). To address this issue, the TGA and DTG curves of pure PEG are also shown in Figure S5. It can be seen that the main weight loss occurred over the temperature range of 110–200 °C, which is in agreement with our claim.

Because the PEG molecules were used initially to achieve exfoliation and expand the layers, the weight loss that occurred up to 500 °C was attributed to the PEG that was trapped within the graphene layers. Also, the second phase of degradation was associated with the decomposition of the graphitic carbon. It is noteworthy that the results suggest that CE-GR had an appropriate degree of functionalization. Also, the weight loss obtained by our method was greater than those reported by conventional methods^25^, suggesting a strong functionalization of carbon nanostructures with organic molecules.

To achieve pure GR, the remaining PEG that was decorated on the structure of CE-GR was removed by thermal treatment under a nitrogen atmosphere at temperatures up to 500 °C for 15 min. Figure 1(d) shows the TGA and DTG curves of the T-GR after freeze drying. As observed in the DTG curve, the only obvious peak was the graphitic peak. Also, the graphitic structure of T-GR remained stable up to 750 °C, indicating that the prepared graphene is totally pure and those functional groups can be removed with a simple and fast method^36^. In addition, the decomposition temperature of both products are same and shifted towards lower temperatures compared to the pure graphite, indicating that the number of layer per flakes decreased.

The Raman spectra of the pristine graphite, CE-GR, and T-GR are shown in Fig. 1, panel (e). A feeble D band can be seen at 1344 cm^–1^, and there are fairly strong G and 2D bands at 1568 and 2695 cm^–1^, respectively. The ratio of the intensities of the D-band to the G-band (I_D_/I_G_) is considered to be the amount of disordered carbon (sp^3^-hybridized carbon) relative to graphitic carbon (sp^2^-hybridized carbon). In functionalization studies of carbon nanostructures, the higher intensity ratio of I_D_/I_G_ indicates the higher disruption of aromatic π-π electrons, implying the partial damage of graphitic carbon produced by expansion and edge functionalization^36^. Although the D bands of all of the samples had weak peaks, the I_D_/I_G_ ratios of CE-GR were relatively higher than those of pristine graphite, which confirmed the successful functionalization via an electrophilic addition reaction under microwave irradiation^37^. Also, the Raman results of CE-GR showed lower I_D_/I_G_ ratios than the graphene oxide and/or chemically-converted graphene^38^, implying that there were fewer defects on the basal planes of the CE-GR.

Also, the poor D-mode (I_D_/I_G_) of T-GR, which was in agreement with the DTG and TGA results, confirmed that the prepared graphene was totally pure and that the functional groups had been removed completely. Surprisingly, the G peak in the spectra of CE-GR and T-GR retained their intensities after the electrophilic addition reaction (insignificant decrease in G mode with CE-GR sample), which confirmed that the quality of the graphene layers that were obtained was preserved. Also, the higher intensity ratio of I(2D)/I(G) confirms the presence of few-layer graphene.

Raman spectroscopy can obviously distinguish a single layer from a bilayer and/or from a few layers by focusing on the shape, size, and intensity of the 2D bands^39^. According to the results of Ferrari *et al*.^39^, as the layer of graphene increases, the 2D band becomes much broader and up-shifted. Accordingly, a considerable change in sizes, shapes, and intensities of the 2D peaks of CE-GR and T-GR are more obvious than they are for the pristine graphite. It can be seen that the 2D bond of the pristine graphite includes a coupled peak, i.e., D_1_ and D_2_ peaks, which produced a broad peak^39^. However, single, sharp 2D peaks were shown in the Raman spectra of CE-GR and T-GR. This change in the 2D bands observed in the CE-GR and T-GR samples potentially verified the presence of the low number sheets.

**Figure 2:**
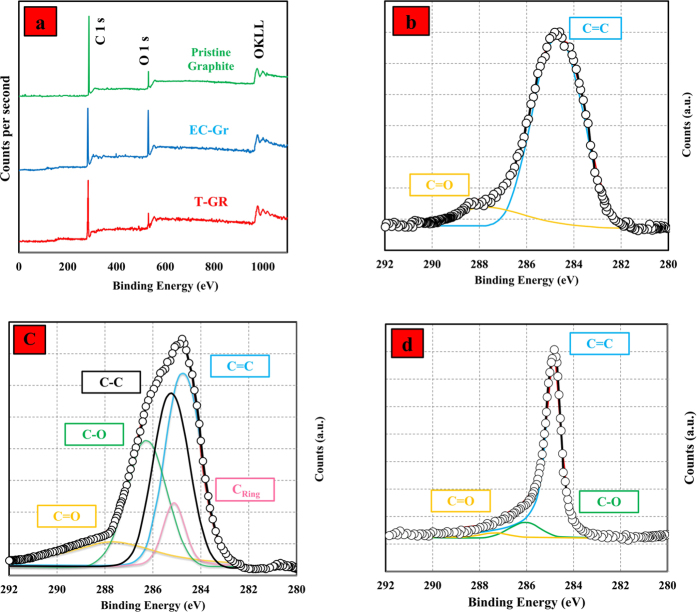
(**a**) XPS survey spectra of CE-GR and T-GR. High-resolution C 1 s spectra of (**b**) pristine graphite, (**c**) CE-GR and (**d**) T-GR.

The natures of pristine graphite, CE-GR, and T-GR were studied by X-ray photoelectron spectroscopy (XPS), as illustrated in Fig. 2a. It can be seen that C 1 s and O 1 s peaks appeared at ~284.5 eV and 531.8 eV, respectively. Based on these results, pristine graphite presents a very small amount of oxygen. Upon functionalization, the intensity of the O 1 s peak increased considerably. It is obvious that PEG functionalities may explain the higher content of oxygen in the CE-GR sample. The amount of oxygen in T-GR decreased after thermal treatment in nitrogen as compared with the CE-GR sample, and, interestingly, it was a bit lower than the amount of oxygen in pristine graphite. The decrease in the O component that was obtained after thermal treatment was associated with the loss of the functional groups between the graphene layers, which was in agreement with the FTIR, TGA, DTG, and Raman results. To investigate the nature of the functional groups, further study was conducted using high-resolution C 1 s scans. Figure 2b–d present the deconvoluted C1s XPS spectra of pristine graphite, CE-GR, and T-GR.

Pristine graphite (Fig. 2b) mainly had a peak around 284.7 eV that corresponded to the C=C network^23^. The peaks corresponded to the sp^3^ C–C is observed at 285.2 eV, indicating the presence of the sp^3^ carbons of PEG units^40^. The minor O component in pristine graphite presents in the form of the C=O group at 287.5 eV. In CE-GR (Fig. 2c), the presence of considerable amount of functional groups was apparent. The peak at ∼ 286.1 eV corresponded to the C–O groups, which resulted from the hydroxyl (C–OH) and/or the epoxide (C–O–C) groups. Clearly the hydroxyl (C–OH) group and the epoxide group had similar C1s binding energies^23^.

The C_ring_ carbon in the furan ring bonded to two hydrogen atoms also was present to a considerable extent in CE-GR at ∼285 eV indicating the functionalization of pristine graphite with PEG molecules. The presence of furan signals could be indicative of the lack of a ring-opening reaction. The C1s XPS spectra of T-GR (Fig. 2d) show a significant decrease in the extent of functional groups. In T-GR, a majority of the functional groups, such as C=O, C_ring_, and C–O, was reduced or removed. Also, in the C 1s spectra, the intensity of the peak corresponding to sp^2^-hydridized carbon (at 284.7 eV) increases continually from CE–GR to T–GR, revealing the effective removal of functional groups from CE–GR and partial restoration of the conjugated graphene sheets^40^. Overall, the above results along with the ratio of I_D/G_ in Raman spectra clearly indicate the strong covalent linkage of PEG moieties to the graphite. Also, the sharp and symmetric C 1s peak of T-GR at 284.8 eV confirmed the presence of a graphitic structure with minimal functional groups, indicating that a large number of covalent-functional groups on CE-GR removed after the thermal treatment^20^. The peak positions and their interpretation are presented in Table 1.

**Figure 3:**
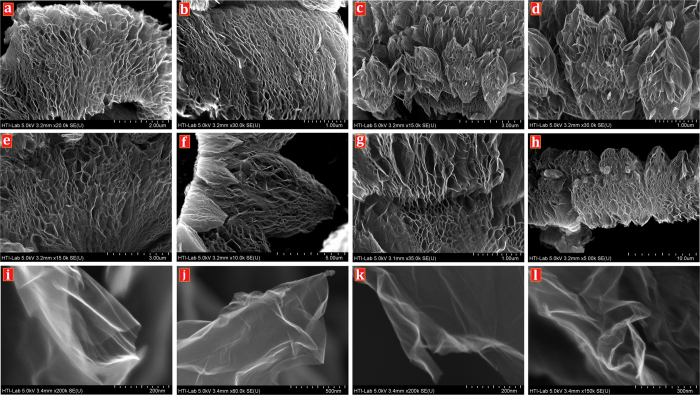
FESEM images of the CE-GR (3**a–h**) and T-GR (3**i–l**).

Figure 3 illustrate the FESEM images of the (a–h) CE-GR and (i–l) T-GR that show a high yield exfoliation was achieved. Note that, for CE-GR samples, high-resolution imaging of FESEM (Fig. 3) was obtained with no pretreatment, such as surface sputtering with a thin gold layer, suggesting that the samples had high conductivity^36^.

Figure 3 shows the exfoliations of the multi-layered graphite into single- or few-layered graphene, which was obtained by microwave-assisted functionalization along with physical cracking by a sonicator, demonstrating a marked reduction in thickness by chemical-mechanical cracking with no significant damage to the size of the grains. The mentioned approach is able to open the graphite layers swiftly, and it produces CE-GR and T-GR with appropriately uniform surfaces.

It is worth mentioning that the FESEM pictures of samples showed some crumpled and curved sheets that were transparent to the electron beam. Also, such a worm-like surface, with crumpled and curved sheets, is due to strict exfoliation and functionalization. Although FESEM images cannot distinguish the precise thickness of flakes and functional groups, the planar morphology of graphene layer, the exfoliation and formation of low-layered graphene are clearly seen from these pictures. It is worth mentioning that FESEM clearly showed intense cleavage surfaces of graphite by functionalization under microwave irradiation and sonication. Some of T-GR images show that the extent of wrinkles, which commonly developed during functionalization and were attributed to the addition of functional groups, almost decreased after thermal treatment. This may be a result of decomposition of functional groups (PEG) during the thermal-treatment procedure. More evidence in this regard is provided by transmission electron microscopy and atomic force microscopy in the following section.

**Figure 4:**
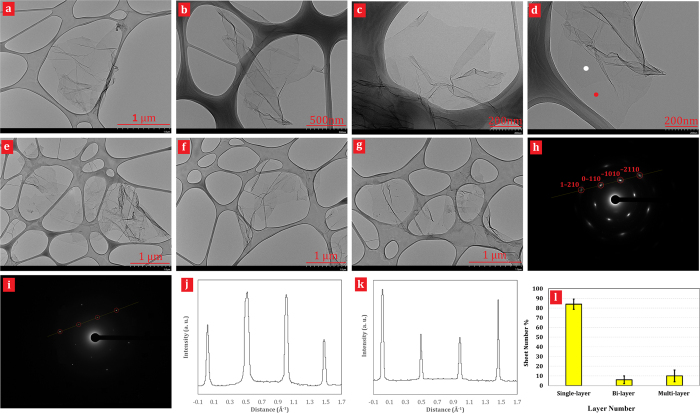
(**a–h**) High-resolution TEM images of CE-GR (**a–c**) and T-GR (**d–g**) graphene. (**h,i**) Electron diffraction patterns taken from the positions of the red (**h**) and white spots (**i**), respectively, of the sheet shown in (**d**) with the peaks labelled by Miller–Bravais indices. The graphene is clearly one layer thick in (**h**) and two layers thick in (**i**). (**j,k**) Diffracted intensity taken along the 1–210 to –2110 axis for the patterns shown in (**h**,**i**) respectively. (**l**) Histogram of the ratios of the intensity of the {1100} and {2110} diffraction peaks for all the diffraction patterns collected.

Figure 4 shows the transmission electron microscopy (TEM) images and select area electron diffraction (SAED) patterns of CE-GR and T-GR. Figure 4a–c show the TEM images of CE-GR, which included some individual graphene sheets with wrinkled morphology and folded edges. It can be seen that large graphene nanosheets (a few hundred square nanometers) resemble crumpled silk veil waves. Figure 4 (panels d–g) illustrate TEM images of the T-GR sample, which comprised of a single/few-layered graphene with large grain sizes. In addition, some of the small and big holes are obvious in Figure S6.

A more definitive identification of graphene can be made by analysis of electron diffraction patterns^41^. For identification of monolayers by electron diffraction, we can use the fact that the ratio of the intensity of the {1100} to the {2110} peaks gives an unambiguous local identification of monolayer, bilayer, and multilayer to provide information on the yield of monolayer graphene. The recent results were reported experimental intensity ratios of I_{1100}_/I_{2110}_ ≈ 0.4 for bilayer graphene and I_{1100}_/I_{2110}_ ≈ 1.4 for monolayer graphene^41^,^42^. Also, we get a bimodal distribution, with peaks centered at I_{1100}_/I_{2110}_ = 0.56 and I_{1100}_/I_{2110}_ = 1.45, representing bilayer and monolayer graphene, respectively, which agreed well with other reported results^22^,^42^. Other quantities are caused by multilayer graphene. As an example of this, Fig. 4d shows what appear to be a graphene monolayer and a graphene bilayer, respectively. Figure 4d is particularly interesting as almost the middle side of the flake consists of at least two layers, whereas on the edge side, a single monolayer protrudes. Figure 4h shows the normal-incidence electron diffraction pattern of the flake in Fig. 4d, taken with beam position close to the red dot. Figure 4i shows normal-incidence selected-area diffraction patterns for the flake in Fig. 4d, taken with beam position close to the white dot. In both cases, the patterns show the typical sixfold symmetry expected for graphite/graphene^43^, allowing us to label the peaks with the Miller–Bravais (hkil) indices. Also, the hexagonal patterns are similar to those in other researches’ reports for single-layer and bilayer graphene.

The main difference between Fig. 4h,i is that for the multilayers (or bilayer) (Fig. 4i), the {2110} spots appear to be more intense relative to the {1100} spots. As mentioned above, this identification of AB stacking in these thin multilayers allows us to differentiate monolayer from bilayer as well as multilayer graphene by inspection of the intensity ratio I_{1100}_/I_{2110}_. To do this, we plot a line section through the (1–210)–(0–110)–(–1010)–(–2110) axis for the patterns in Fig. 4i in Fig. 4k, respectively. In Fig. 4j, we see that the inner peaks, (0–110) and (–1010), are more intense than the outer ones, (1–210) and (–2110), confirming that the region marked by the red dot in Fig. 4d is monolayer. Conversely, Fig. 4k shows inner peaks that are less intense than the outer ones, confirming that the area around the white dot in Fig. 4d consists of more than one layer. By analysing a large number of TEM images and their electron diffraction patterns based on the intensity ratio I_{1100}_/I_{2110}_, paying close attention to the uniformity of the flake edges, we can generate flake thickness statistics as shown in Fig. 4l. The distribution of the number of layers per flakes was obtained by analyzing 3 batches of flakes (first group 97 flakes, second group 50 flakes and third group 50 flakes). The results suggested that about 84% and 6% of the sheets were single- and bi-layered, respectively (Fig. 4l). Also, we rarely (almost 10% of flakes) observe large objects with thickness of more than a few layers. Thus, we believe that, graphite has been extensively exfoliated to give monolayer as is obvious in FESEM images as well.

**Figure 5:**
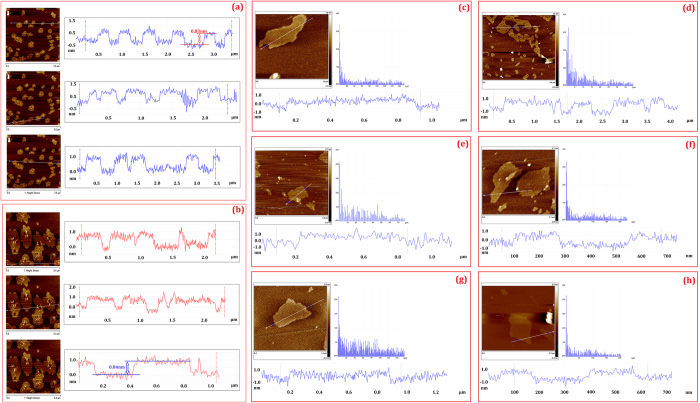
AFM ichnography and cross-section contour of (**a,c–e**) CE-GR and (**b,f–h**) T-GR.

AFM was utilized for the further morphological characterization of the thin-layered graphene and for the investigation of the thicknesses of the CE-GR and T-GR flakes. AFM samples were prepared by sonicating (two minutes in a bath sonicator) CE-GR and T-GR sheets in DMF without any additives, respectively. Figure 5 and Figure S7 present typical AFM images in which no graphene sheets were identified as being layered over other sheets, and there were more than 50 and 20 flakes (Fig. 5 and Figure S7) in CE-GR and T-GR images, respectively, and, interestingly, all of the sheets were single-layer sheets. As shown in Fig. 5 and Figure S7, both samples indicated that all of the sheets had thicknesses of less than 1 nm. With a maximum height of 1 nm in both figures, the CE-GR and T-GR samples showed the thickness of one layer, which some of them was decorated with many large and small holes and it is attributed to severe side functionalization by the electrophilic aromatic substitution under microwave irradiation. Also, some of flakes shows no big holes. It is worth to note that for energy applications, some defects or functional groups can be beneficial by representing high specific surface area.

To investigate the effects of functionalization and thermal treatment on the specific surface area of CE-GR and T-GR, N_2_ adsorption–desorption isotherms were measured by a surface area analyzer (Quantachrome Autosorb-1 analyzer at 77 K). The N_2_ adsorption–desorption isotherms of the CE-GR and T-GR are shown in Figure S8 and Table 2. The nitrogen adsorption isotherms exhibit a mixture of type IV and type V with a pronounced hysteresis at a high relative pressure region, implying the presence of a large number of mesopores in both samples. In addition, CO_2_ adsorption-desorption was performed to assess micropores (pores less than 1 nm) in the low-pressure region. BET analysis indicated that the specific surface area of CE-GR and T-GR were up to 761 and 1559 m^2^/g, respectively. Also, the pores in T-GR have a well-defined micro-mesopore size distribution as shown in Table 2, with a huge increase in pore volume (up to 4.53 cm^3^/g) relative to CE-GR. This larger specific surface area of T-GR suggests that the introduction of PEG between the 2D graphene sheets can increase the layer-to-layer stacking to a greater extent than T-GR or the filled the porous area.

**Figure 6:**
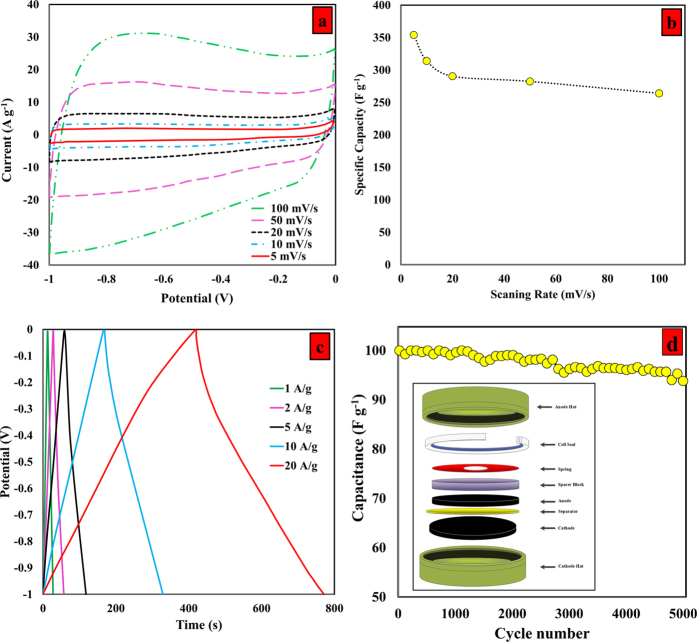
(**a**) The CV curves at different scan rates, (**b**) the specific capacitance versus scan rate, (**c**) galvanostatic charge/discharge curves at the different current densities of T-GR, and (**d**) Capacitance retention ratio as a function of the potential sweep rates for T-GR supercapacitors up to 5,000 cycles. Inset: A configuration of a test cell for electrochemical measurement.

Figure 6 shows the electrochemical properties of the T-GR that were obtained with the organic and aqueous systems. After synthesizing highly-porous, single-layered graphene, the performance of the T-GR supercapacitor was investigated by a symmetrical, two-electrode system in an organic electrolyte. Figure 6a shows the CV of the highly-porous, single-layered graphene supercapacitor in 6-M KOH as an aqueous system at different scan rates. It can be seen that all CV curves at the different scan rates presented a rectangular shape, which is indicative of excellent charge propagation at the electrode surface as well as following the electric double layer capacitive properties of T-GR. The specific capacitance of T-GR was obtained by calculating the integrated area of CV, as observed in the Supplementary information. The specific capacitance of T-GR for different scanning rates is shown in Fig. 6b. It is seen that the specific capacitance decreased as the scanning rate increased. When the scanning rate increased to its maximum value of 100 mV/s, the capacitance still presented a fairly high extent of 264 F/g, almost 75% of the maximum capacitance of 354 F/g at the scanning rate of 5 mV/s. Compared with previous studies of the graphene family summarized in Table S1 23,27,36,44,45,46,47,48,49,50, the T-GR with a capacitance of 354.1 Fg^−1^ at 5 mVs^−1^, exhibits excellent performance as a supercapacitor.

Figure 6c shows the galvanostatic charge/discharge curve of the T-GR supercapacitor in a 1.2M MeEt_3_NBF_4_(AN) electrolyte solution for various current densities. It is seen that the anodic charging curves were reasonably symmetrical with the corresponding cathodic discharging curves, which was in agreement with the CV results. This is indicated of significant capacitive reversibility of T-GR. These results suggest that the specific capacitance of T-GR decreases as the charge/discharge rate increases, indicating good rate capability of the T-GR supercapacitor. Also, the initial decrease in the voltage of the discharge curves was insignificant, confirming an insignificant equivalent series resistance (ESR) in the symmetric supercapacitor; thus, T-GR has a promising potential for use in high-power operations.

Also, the electrochemical stability of T-GR in the presence of organic electrolytes was assessed via the galvanostatic charge–discharge method at 1.0 A g^−1^ (Fig. 6d). Interestingly, the T-GR supercapacitor retained 93.8% of its initial specific capacity, which again highlights the promising potential of T-GR in terms of the electrochemical stability and degree of reversibility. The specific capacitance was up to 271 F/g throughout the cycle numbers from 0 to 5000, indicating a significant power capability.

In agreement with the previous results presented in Table S1, the higher extent of capacity in the presence of the aqueous electrolytes than that of the organic electrolytes was reasonable and has been reported numerous times^50^,^51^. Also, a comparison of previous results and the results of this study confirmed that the special structure of highly-porous, single-layered graphene electrodes considerably improved the specific surface area of sheets and thus the specific capacitance.

## Conclusions

Single-layered graphenes were produced by a potentially industrially-scalable, cost-effective, liquid-phase exfoliation in the presence of chemical-mechanical treatment. First, graphite-polyethylene glycol (PEG) was synthesized by covalent functionalization under microwave irradiation to enhance its dispersibility in polar aprotic organic solvents. Few-layer graphenes were obtained from PEG-treated graphite. Functionalization was confirmed by characterization instruments, and, after exfoliation, the statistical studies concerning the distribution of the number of layers indicated that about 90% of few-layered graphene had one and two layers, about 84% of which were single-layered. The few-layered graphenes illustrated promising potential in the storage of electrochemical energy. The highly-porous, single-layered structure of graphene provides a special condition for electrodes due to the specific surface area of the sheets. The nanoscopic porous morphology of single-layered, graphene-based electrodes have an important role in increasing BET surface as well as the specific capacitance of graphene.

## Additional Information

**How to cite this article**: Amiri, A. *et al*. Mass production of highly-porous graphene for high-performance supercapacitors. *Sci. Rep.* **6**, 32686; doi: 10.1038/srep32686 (2016).

## Supplementary Material

Supplementary Information

## Figures and Tables

**Table 1:** Peak positions of various groups in C1s Spectra of pristine graphite, CE-GR and thermally-treated graphene.

sample	functional group peak positions (eV)				
	C=C	C-C	C-O	C=O	C_ring_
Pristine Graphite	284.7	—	—	287.7	
CE-GR	284.6	285.2	286.1	287.6	285
T-GR	284.8	—	286.1	287.3	—

**Table 2:** Pore structure of the CE-GR and T-GR.

Sample	S_BET_ (m^2^ g^−1^)	pore volume (cm^3^ g^−1^)		
		Micropore	Mesopore	Total
CE-GR	761	0.77	2.15	2.92
T-GR	1559	0.92	3.61	4.53
