# Supplementary material for: Mass production of highly-porous graphene for high-performance supercapacitors
Source: Sci Rep. 2016 Sep 8;6:32686. doi: 10.1038/srep32686 (PMC5015014; doi:10.1038/srep32686)
Supplement: Supplementary Information [file srep32686-s1.pdf]

Supplementary Information

Mass production of highly-porous graphene for high-performance supercapacitors

Ahmad Amiri [1]*, Mehdi Shanbedi [2]*, Goodarz Ahmadi [3]*, Hossein Eshghi [4], S.N. Kazi [1]*, B.T. Chew [1], Maryam Savari [5], Mohd Nashrul Mohd Zubir[1]

[1] Department of Mechanical Engineering, University of Malaya, Kuala Lumpur, Malaysia,

[2] Department of Chemical Engineering, Faculty of Engineering, Ferdowsi University of Mashhad, Mashhad, Iran,

[3] Department of Mechanical and Aeronautical Engineering, Clarkson University, Potsdam, NY 13699, USA.

[4] Department of Chemistry, Faculty of Science, Ferdowsi University of Mashhad, Mashhad, Iran.

[5] Faculty of Computer Science and Information Technology, University of Malaya, Kuala Lumpur, Malaysia.

* Corresponding authors' Email:

gahmadi@clarkson.edu (G. Ahmadi)

mehdi.shanbedi@stu-mail.um.ac.ir (M. Shanbedi)

ahm.amiri@gmail.com (A. Amiri)

salimnewaz@um.edu.my (S. N. Kazi)

Chemicals.

All reagents such as Tetrahydrofurfuryl polyethylene glycol ((PEG, C_5H_9O $(C_2H_4O)_{n=2,3}OH$, MW~200), $AlCl_3$, N,N-dimethylformamide (DMF), N,N-Dimethylacetamide (DMA), g-butyrolactone (GBL), ethylene glycol (EG), polytetrafluoroethylene (PTFE) binder and HCl with analytical grade were obtained from Sigma-Aldrich and were employed without additional purification. The natural graphite with 100 mesh (<150 μm) and purity of 99.9% was obtained from Alfa Aesar. Figure S_1 is shown the experimental procedure for functionalization as well as exfoliation of graphite and producing CE-GR and T-GR.

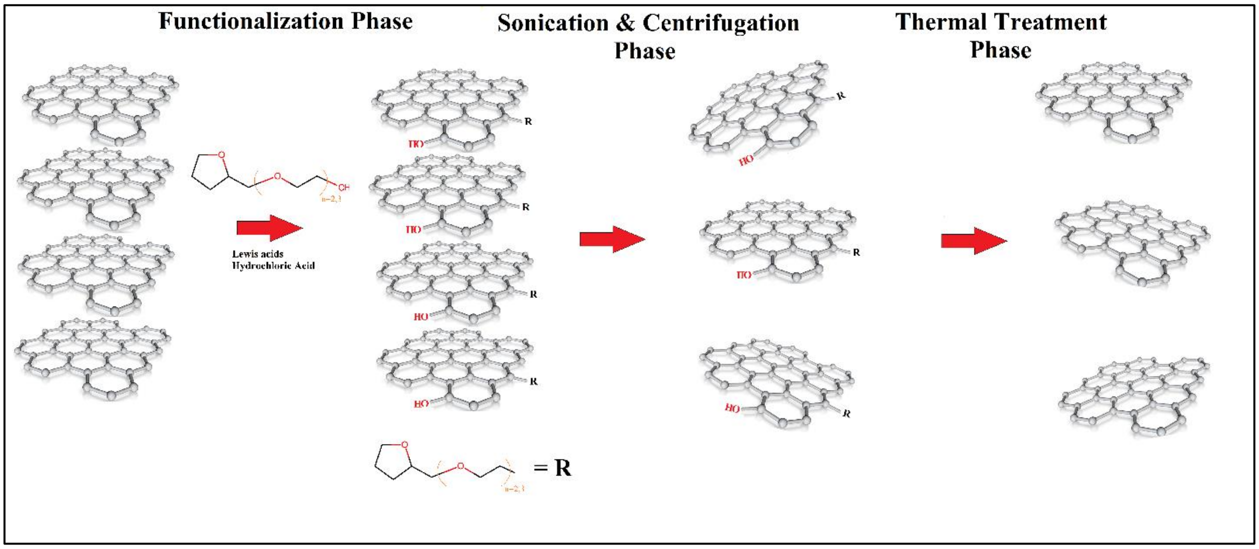

Figure S_1. The experimental procedure for functionalization as well as exfoliation of graphite and synthesizing CE-GR and T-GR.

Based on the results, the functionalized graphite was expanded and was much more soluble in DMF, DMA, g-butyrolactone (GBL) and EG than the pristine graphite, as shown in Figure S_2 panel 1. It is noteworthy that a majority of flakes has remained stable for more than 1 week. The easily-miscible PEG functionalities may explain the higher dispersion of the functionalized and expanded graphite. For preparing pure graphene without functional groups, thermal treatment under nitrogen atmosphere up to 500 °C have been applied for 15 min to remove all PEG molecules or other impurities and product labeled as T-GR. The extracted black

pure graphene powder (Figure S2 panel 2) has a big visual difference with the shiny metallic grey of the pristine graphite.

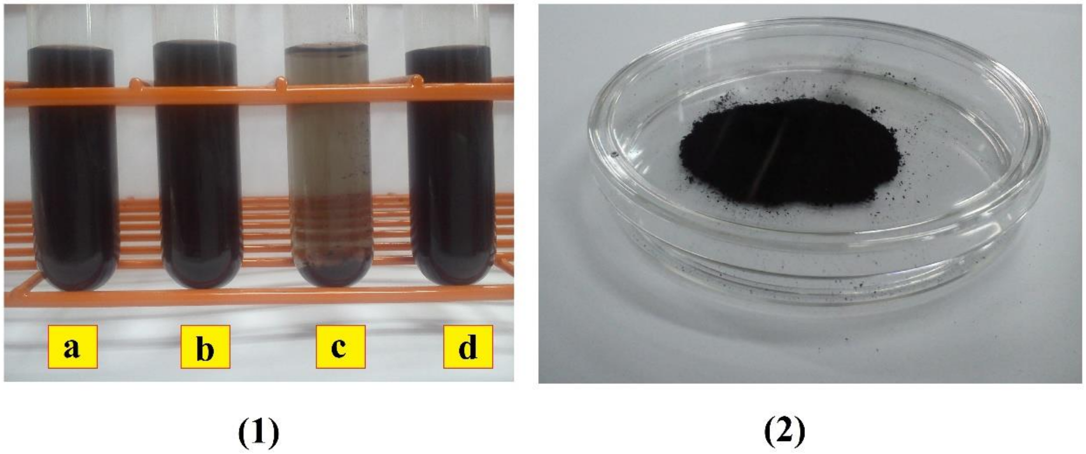

Figure S2. (1) Photographs of functionalized and expanded graphite dispersed in (a) DMF, (b) DMA, (c) g-butyrolactone and (d) EG. (2) The extracted black pure graphene powder (T-GR).

Characterizations.

Midinfrared spectra of CE-GR were evaluated by a Fourier transform infrared spectroscopy (Bruker) in the region 400–4000 cm^{-1} on KBr pellets. To investigate functionalization and layers changes, Raman spectroscopy (Renishaw confocal spectrometer) was employed at 514 nm at room temperature. The thermogravimetric analysis was measured by a Perkin Elmer TGA-7 in the atmosphere of air. The weight loss of CE-GR, T-GR and pristine graphite were analyzed in TGA as a function of temperature at the heating rate of 10 °C/min up to a temperature of 900 °C under 50 cm^3/min flow rate of air.

In order to study surface morphology of the CE-GR and T-GR, field emission scanning electron microscopy (FESEM SU8000 Series UHR Cold-Emission) and transmission electron microscopy (HT7700 120kV High-Contrast/High-Resolution Digital TEM) were applied. The preparation of TEM samples comprises of sonication of CE-GR or T-GR sample in ethanol and followed by depositing one drop of the aforementioned suspension on a lacey carbon grid and allowed to dry. An ultrasonic processor (Misonix Inc., Farmingdale,

New York, NY, USA) with output of 600 W was used. All samples sonicated with 85% of total power of ultrasonic processor. Also, a Milestone MicroSYNTH programmable microwave system equipped with temperature controller was employed to perform the process of functionalization.

To investigate more in morphology of CE-GR and T-GR samples, atomic force microscopy, AFM, (Bruker) were utilized. AFM observation was conducted for the slightly sonicated CE-GR and T-GR samples on the freshly cleaved mica surfaces (ScanAsyst mode, frequency 1 Hz, Bruker). In order to provide TEM sample, CE-GR after drying was first dispersed in ethanol, and then dropped on a lacey carbon grid and followed by drying under vacuum condition.

Colloidal stability

Plot of absorbance versus wavelength for various colloidal solutions were studied to trace the presence of specific particle within the binary system. Figure S3 (a-d) shows the plot of absorbance intensity versus wavelength for expanded graphite in EG, DMA, DMF and GBL taken at specific period. The measurements were performed after 24 hrs of sample preparation to remove any non-reacted negligibly charged colloids which rapidly agglomerated and settled at the bottom of the sampling bottle. First, the spectral results suggested that the colloidal mixtures of PEG-treated graphite-solution exhibit similar trend as to the single absorbance spectral curve of PEG. This observation verified the fact that PEG remain within the colloidal mixture, implying successful functionalization.

Unsurprisingly, the functionalized graphite with PEG could easily disperse in EG, DMA, DMF and GBL solvents, respectively. Figure S3 depicts the significant dispersibility of PEG-treated graphite in different solutions.

To investigate the colloidal stability of PEG-treated graphite in different solutions, further measurement was conducted to observe the chronological progress of the suspension's light absorbance with respect to time, which is illustrated based on relative concentration in Figure S3(e). The measurement was carried out at peak wavelength of each material to trace the alteration in the intensity which can be further used to describe the suspension stability at the constant weight fraction of PEG-treated graphite. It can be seen that all colloidal mixtures show a downward trend of relative concentration as the time progressed, indicating that the level of particle concentration and thus the stability subsided. Also, the relative concentration (absorbance intensity)

including PEG-treated graphite/EG shows the minimum amount of sediment. The easily-miscible PEG functionalities in EG may explain the higher dispersion of the functionalized graphite as compared with others.

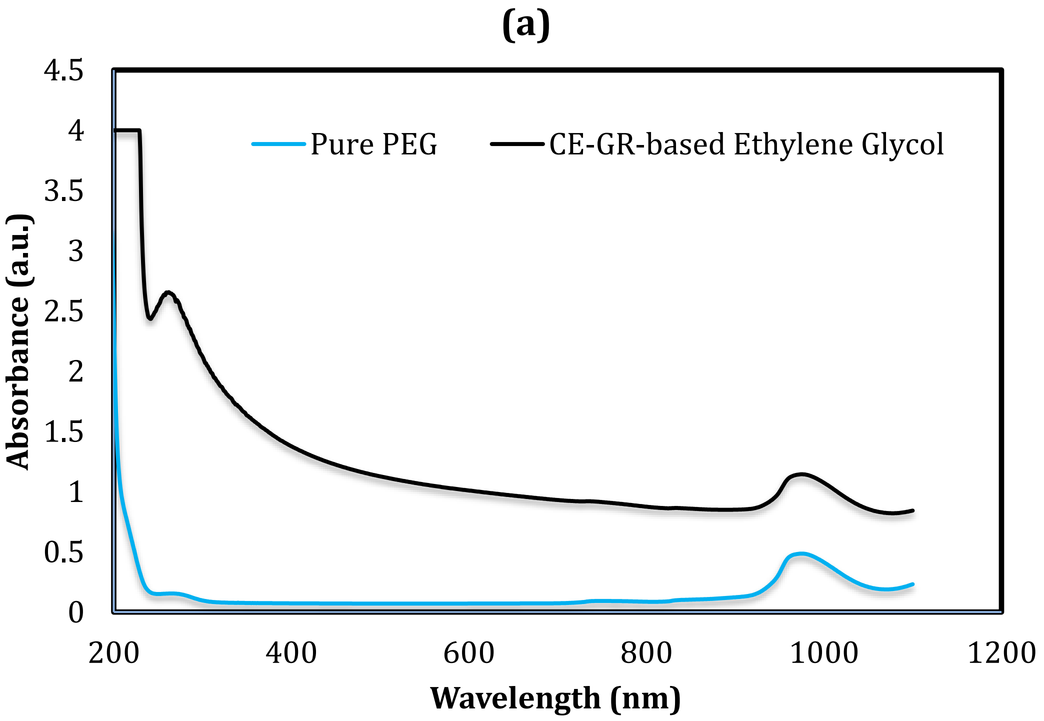

(b)

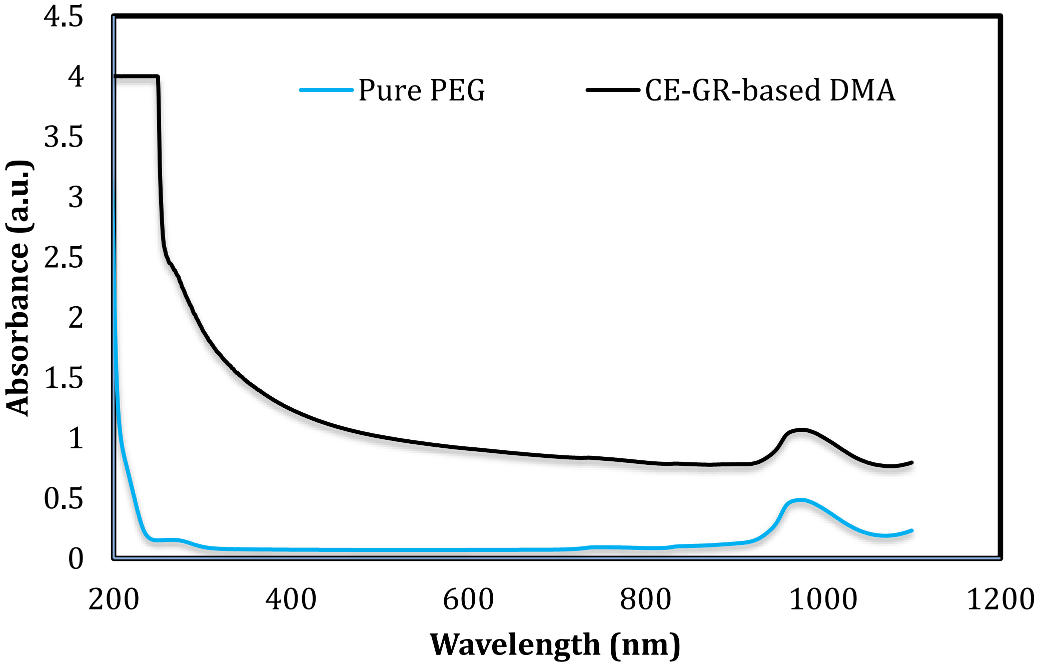
Pure PEG
CE-GR-based DMA
Absorbance (a.u.)
4.5
4
3.5
3
2.5
2
1.5
1
0.5
0
200
400
600
800
1000
1200
Wavelength (nm)

(c)

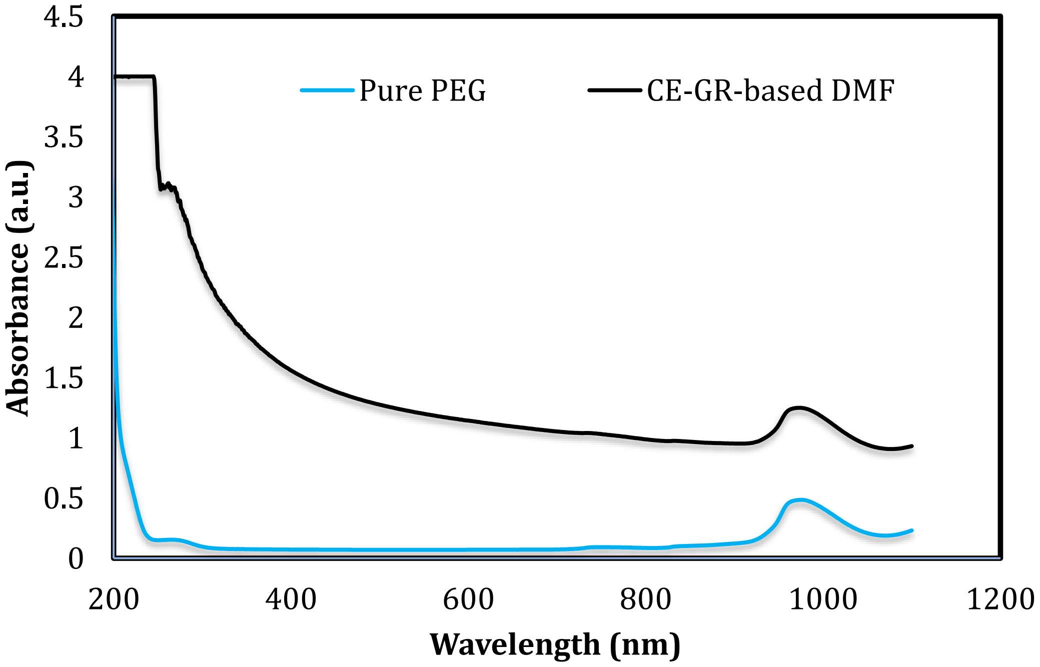
Pure PEG
CE-GR-based DMF
Absorbance (a.u.)
4.5
4
3.5
3
2.5
2
1.5
1
0.5
0
200
400
600
800
1000
1200
Wavelength (nm)

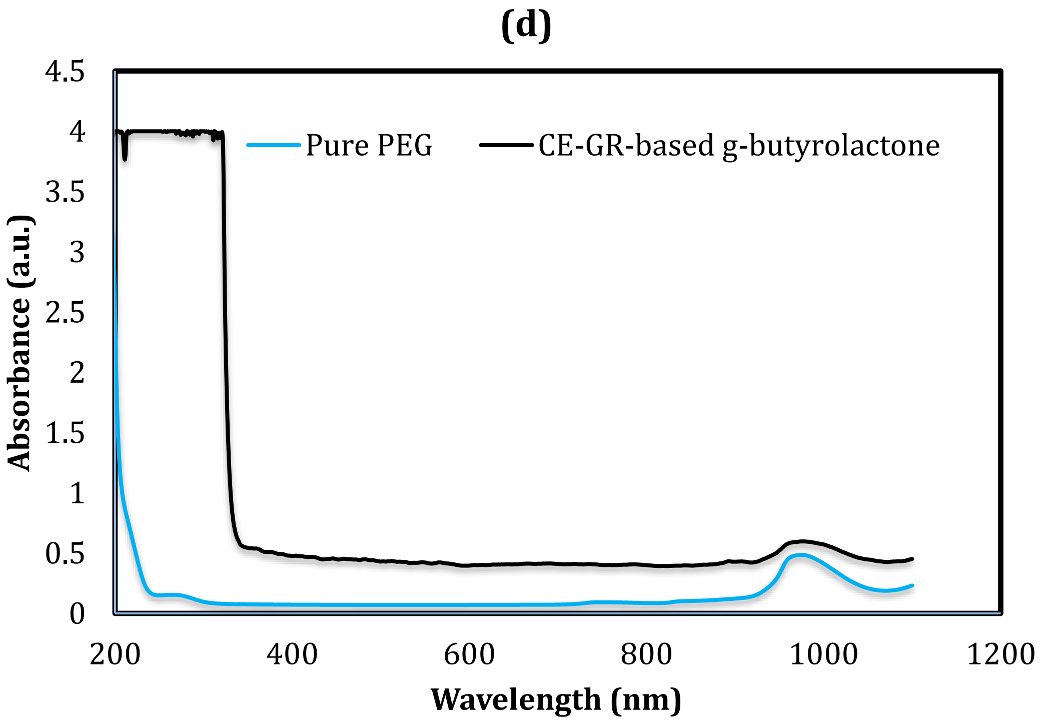

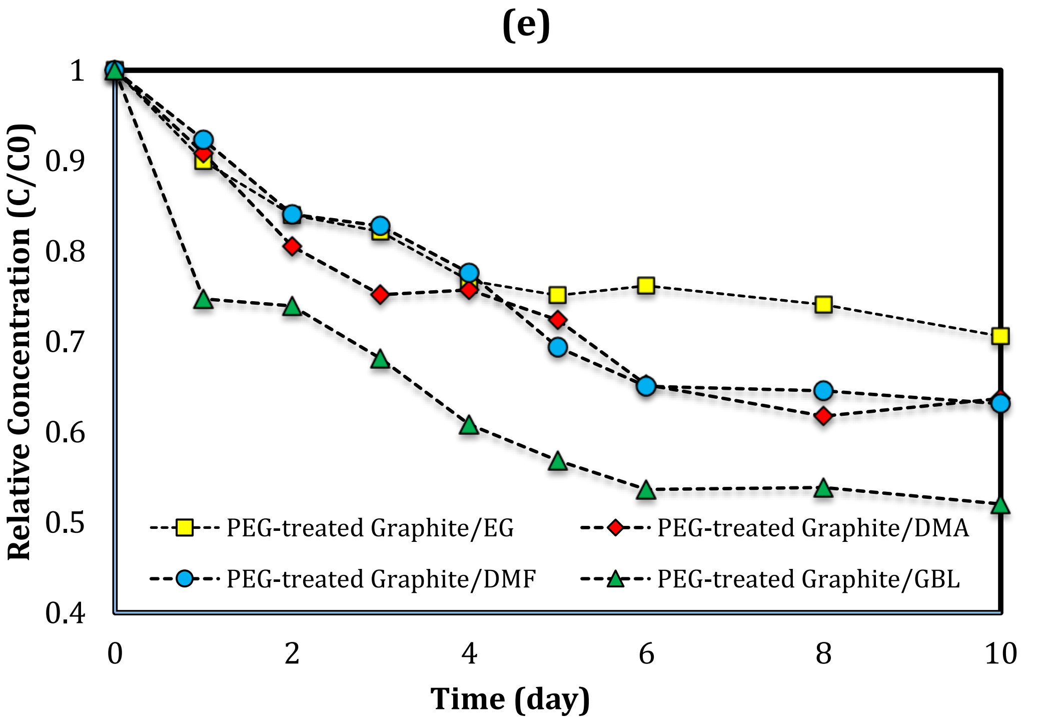

Figure S3. Plot of absorbance versus wavelength for pure PEG and (a) PEG-treated graphite/EG, (b) PEG-treated graphite/DMA, (c) PEG-treated graphite/DMF, (d) PEG-treated graphite/GBL and (e) plot of colloidal stability of PEG-treated graphite in EG, DMA, DMF and GBL.

Electrochemical preparation and Measurements.

To prepare electrodes, a certain amount of highly porous single-layered graphene (T-GR) was mixed with 10 wt% PTFE and then ethanol was dropwise added. Electrode preparation was followed by 30 min sonication and dried. The obtained homogeneous paste was coated on the foam (nickel) and dried quickly at 110 °C. To obtain homogenous electrodes in terms of coated materials, this procedure was repeated 5 times and the final electrodes were cold-pressed at 3 MPa. To eliminate ethanol and moisture, a 48 hr drying at 100 °C in vacuum condition was applied. Two obtained electrodes including highly porous single-layered graphene, spring and a porous separator (Figure S4) were the main parts of two-electrode test cell.

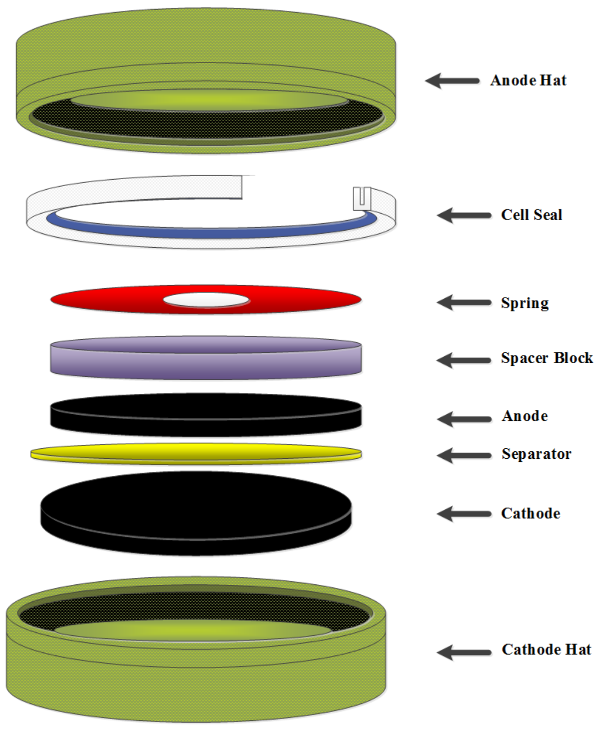

Figure S4. (a) A configuration of a test cell for electrochemical measurement.

Also, the specific capacitance was calculated based on Eq. (1). That is:

$$C_p = 2\int (IdV)/mv\Delta V \quad (1)$$

where I, V, ν and m are the current, potential, scan rate and mass of electrode material. Also, the specific capacitances of highly porous single-layered graphene were obtained from Galvanostatic charge-discharge measurements using Eq. (2).

$$C_p = 4I\Delta t/m\Delta V \quad (2)$$

where C_p and ∆t represent the specific capacitances and the discharging period[1].

TGA-DTG of pure PEG

To better understand the evolution into CE-GR, the TGA and DTG curves of pure PEG are also show in Figure S5. As mentioned, CE-GR illustrates a mild weight loss in the temperature range of 100–225 °C, which was due to the decomposition of the covalently-grafted organic addends. The thermogravimetric analysis was carried out on the pure PEG to investigate its thermal properties. The main weight loss occurred over the temperature range of 100-200 °C, which confirms our claim.

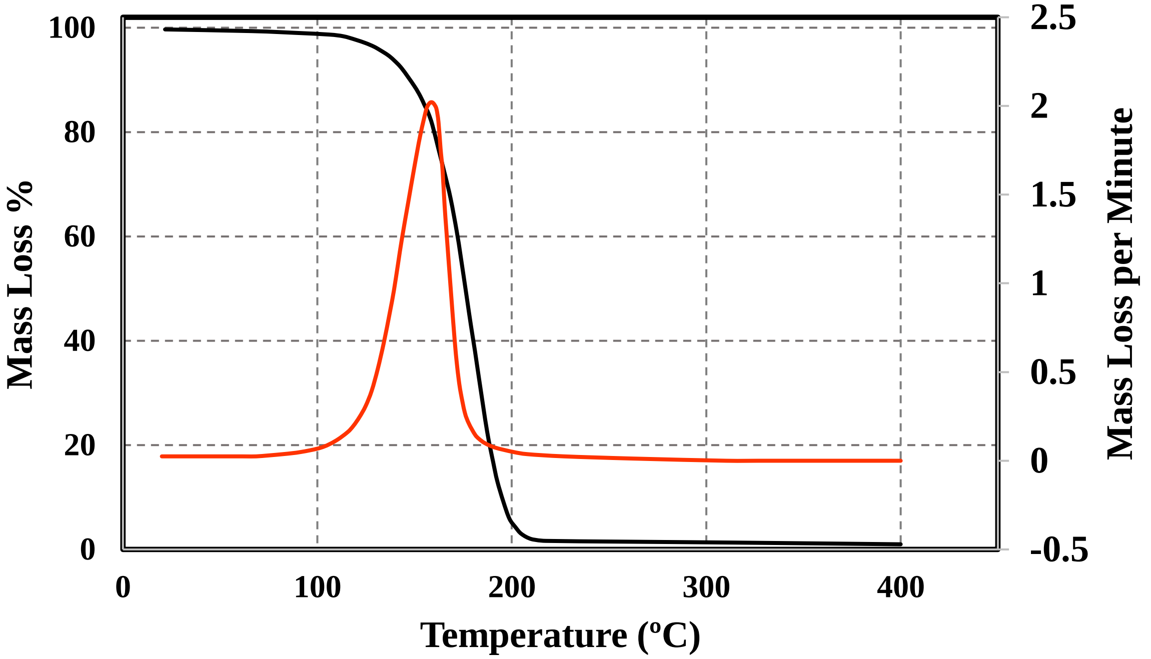

Figure S5. TGA and DTG curves of pure PEG.

TEM

It can be seen in TEM images that most of the samples are single layered. Although some of them have holes, a majority of them does not have big holes. It can confirm that the functionalization does not happen only at the graphene edges, but edge is more possible for functionalization. The presence of hole can be attributed to the electrophilic addition reactions between poly ethylene glycol (PEG) and graphene through a microwave-assisted method. When carbon nanostructures are exposed to microwaves, strong absorptions are obtained, which producing intense heating and thus providing a suitable condition for doing reaction. Also, an active electrophilic reagent formed by protonation of the PEG can speed the electrophilic aromatic substitution[2, 3, 4, 5].

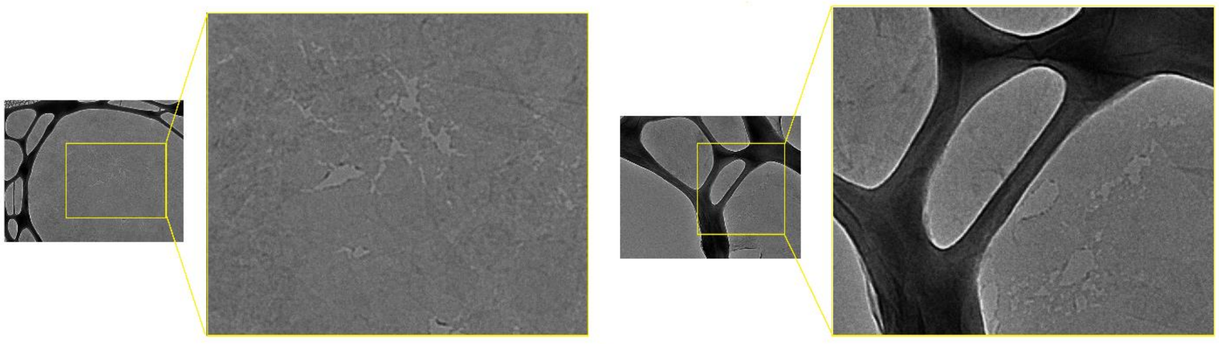

Figure S6. TEM images of the CE-GR.

Atomic Force Microscopy.

As shown in Figure S7, both samples indicate that all of the sheets are of the thickness below 1 nm. With a maximum height of 1 nm in Figure S7, the CE-GR and T-GR samples show the thickness of 1 layer, which some of them were decorated with so many small and big holes.

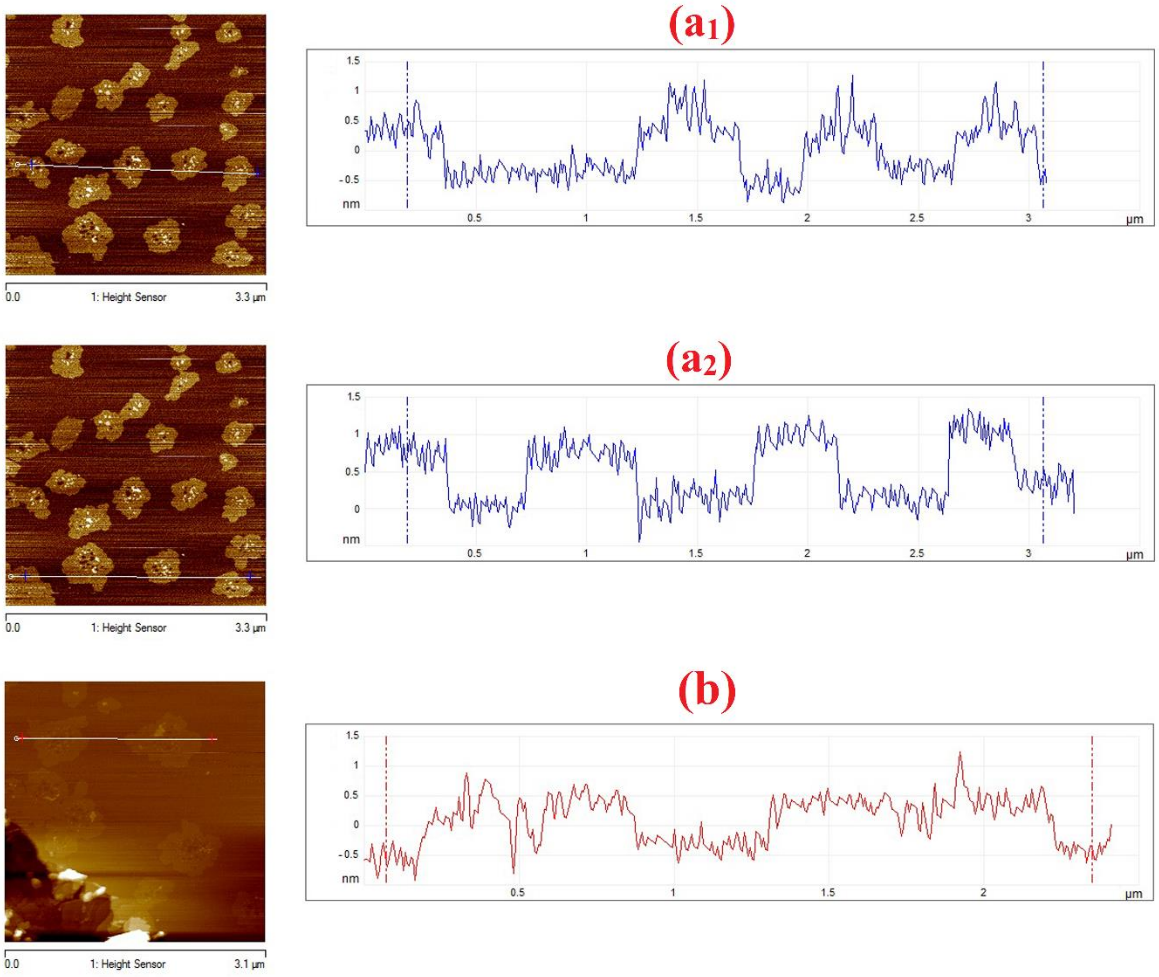

Figure S7. AFM ichnography and cross-section contour of ($a_{1\&2}$) CE-GR and (b) T-GR.

N_2 adsorption–desorption

The N_2 adsorption–desorption isotherms of the CE-GR and T-GR are shown in Figure S8 and Table 1. It can be seen that the N_2 adsorption amount of the T-GR was higher than that of CE-GR. While the specific surface area

of T-GR after annealing at high temperature of 500 °C was 1559 m² g⁻¹, the respective surface areas of the CE-GR was just 761 m² g⁻¹. This larger specific surface area of T-GR suggests that the introduction of PEG between the 2D graphene sheets can increase the layer-to-layer stacking, when compared to that of T-GR or filled the porous area.

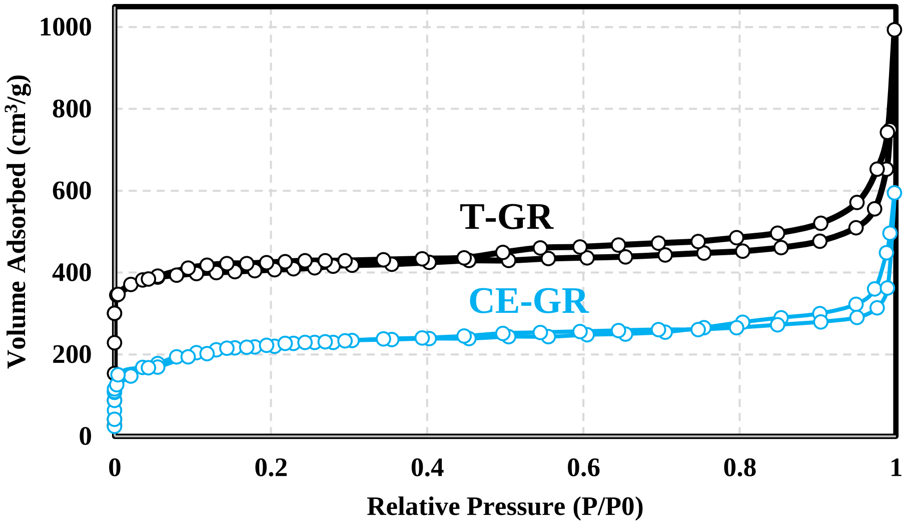

Figure S8. N_2-adsorption/desorption of the CE-GR and T-GR

Table S1. Surface area/pore volume and capacitance values of graphene-based supercapacitors of the different studies

Graphene material	Bet surface area (m^2 g^{-1})	pore volume (cm^3 g^{-1})	Specific capacitance (F g^{-1})	Scan rate (mV s^{-1})	Reference
**Crumpled nitrogen-doped graphene nanosheets-900**	465.0	3.42	302.0 (KOH) 248.4 (Organic)	5	Ref [6]

**Crumpled nitrogen-doped graphene nanosheets-700**	431.7	2.89	300.4 (KOH)<br>237.2 (Organic)	5	Ref [6]
**Reduced graphene**	408.6	0.52	146.8 (KOH)<br>107.0 (Organic)	5	Ref [6]
**Thermally reduced graphene sheets**	76.3	0.05	69.3(KOH)<br>51.7 (Organic)	5	Ref [6]
**Microwave-expanded graphite oxide**	3100	2.14	200.0 (Organic)	100	Ref [7]
**Chemically reduced graphite oxide**	705.0	---	100 (KOH) /<br>99 (Organic)	20	Ref [8]
**Chemically reduced graphite oxide**	320	---	205 (KOH)	---	Ref[9]
**Microwave expanded graphite oxide**	463	---	191 (KOH)	10	Ref[10]
**Thermally reduced graphite oxide**	---	---	122 (Organic)	5	Ref[11]
**Thermally expanded graphite oxide**	925	---	117 (H_2SO_4)<br>75 (Ionic liquid)	100	Ref[12]
**Thermally expanded graphite oxide**	368	---	264 (KOH) /<br>122 (Organic)	10	Ref[13]

**Nitrogen-doped graphene**			282 (KOH) / 223 (Organic)	---	Ref[14]
**Chemically reduced graphene oxide**	615.2	---	110.4	---	Ref[15]
**N-doped graphene sheets**	630.6	---	144.9	---	Ref[15]
**Highly porous single-layered graphene**	1559	4.53	354 (KOH) 289 (Organic)	5	This work
**Highly porous single-layered graphene**	1559	4.53	264 (KOH) 232 (Organic)	100	This work

References

1. Wang R, Yan X. Superior asymmetric supercapacitor based on Ni-Co oxide nanosheets and carbon nanorods. *Sci Rep* **4**, (2014).

2. Singh MS. *Advanced organic chemistry: reactions and mechanisms*. Pearson Education India (2004).

3. Olah GA, Germain A, Lin HC, Forsyth DA. Electrophilic reactions at single bonds. XVIII. Indication of protosolvated de facto substituting agents in the reactions of alkanes with acetylium and nitronium ions in superacidic media. *Journal of the American Chemical Society* **97**, 2928-2929 (1975).

4. Vázquez E, Prato M. Carbon nanotubes and microwaves: interactions, responses, and applications. *Acs Nano* **3**, 3819-3824 (2009).

5. Lin W*, et al.* Microwave makes carbon nanotubes less defective. *ACS nano* **4**, 1716-1722 (2010).

6. Wen Z*, et al.* Crumpled Nitrogen-Doped Graphene Nanosheets with Ultrahigh Pore Volume for High-Performance Supercapacitor. *Advanced Materials* **24**, 5610-5616 (2012).

7. Zhu Y*, et al.* Carbon-based supercapacitors produced by activation of graphene. *Science* **332**, 1537-1541 (2011).

8. Stoller MD, Park S, Zhu Y, An J, Ruoff RS. Graphene-based ultracapacitors. *Nano letters* **8**, 3498-3502 (2008).

9. Wang Y*, et al.* Supercapacitor devices based on graphene materials. *The Journal of Physical Chemistry C* **113**, 13103-13107 (2009).

10. Zhu Y, Murali S, Stoller MD, Velamakanni A, Piner RD, Ruoff RS. Microwave assisted exfoliation and reduction of graphite oxide for ultracapacitors. *Carbon* **48**, 2118-2122 (2010).

11. Zhu Y*, et al.* Exfoliation of graphite oxide in propylene carbonate and thermal reduction of the resulting graphene oxide platelets. *Acs Nano* **4**, 1227-1233 (2010).

12. Vivekchand S, Rout CS, Subrahmanyam K, Govindaraj A, Rao C. Graphene-based electrochemical supercapacitors. *Journal of Chemical Sciences* **120**, 9-13 (2008).

13. Lv W*, et al.* Low-temperature exfoliated graphenes: vacuum-promoted exfoliation and electrochemical energy storage. *ACS nano* **3**, 3730-3736 (2009).

14. Jeong HM*, et al.* Nitrogen-doped graphene for high-performance ultracapacitors and the importance of nitrogen-doped sites at basal planes. *Nano letters* **11**, 2472-2477 (2011).

15. Qiu Y, Zhang X, Yang S. High performance supercapacitors based on highly conductive nitrogen-doped graphene sheets. *Physical Chemistry Chemical Physics* **13**, 12554-12558 (2011).
